# Association between CYP2D6 phenotype and recurrence of *Plasmodium vivax* infection in south Korean patients

**DOI:** 10.1186/s12936-022-04311-6

**Published:** 2022-10-10

**Authors:** Sungim Choi, Heun Choi, Seong Yeon Park, Yee Gyung Kwak, Je Eun Song, So Youn Shin, Ji Hyeon Baek, Hyun-IL Shin, Shin-Hyung Cho, Sang-Eun Lee, Jeong-Ran Kwon, Sookkyung Park, Miyoung Kim, Hong Sang Oh, Yong Chan Kim, Min Jae Kim, Joon-Sup Yeom

**Affiliations:** 1 Division of Infectious Diseases, Department of Internal Medicine, Dongguk University Ilsan Hospital, Goyang-si, Gyeonggi-do Republic of Korea; 2 Department of Infectious Diseases, National Health Insurance Service Ilsan Hospital, Goyang-si, Gyeonggi-do Republic of Korea; 3 Division of Infectious Diseases, Department of Internal Medicine, Inje University Ilsan Paik Hospital, Goyang-si, Gyeonggi-do Republic of Korea; 4 Department of Internal Medicine, International St. Mary’s Hospital, Catholic Kwandong University College of Medicine, Incheon, Republic of Korea; 5 Department of Internal Medicine, Inha University College of Medicine, Incheon, Republic of Korea; 6 Division of Vectors and Parasitic Diseases, Bureau of Infectious Disease Diagnosis Control, Korea Disease Control and Prevention Agency, Chungcheongbuk-do, Republic of Korea; 7 Division of Zoonotic and Vector borne Disease Control, Bureau of Infectious Disease Policy, Korea Disease Control and Prevention Agency, Chungcheongbuk-do, Republic of Korea; 8 Department of Internal Medicine, Armed Forces Capital Hospital, Seongnam-si, Gyeonggi-do Republic of Korea; 9 Division of Infectious Diseases, Department of Internal Medicine, Yongin Severance Hospital, Yonsei University College of Medicine, Yongin-si, Republic of Korea; 10 Department of Infectious Diseases, Asan Medical Center, University of Ulsan College of Medicine, Seoul, Republic of Korea; 11 Division of Infectious Diseases, Department of Internal Medicine, Severance Hospital, Yonsei University College of Medicine, Seoul, Republic of Korea

**Keywords:** CYP2D6, Primaquine, Relapse, *Plasmodium vivax*

## Abstract

**Background:**

Primaquine is activated by CYP2D6 in the hepatocytes. In Korea, primaquine is the only hypnozoitocidal agent used for patients with vivax malaria. Thus, patients with poor CYP2D6 activity could have an increased risk of primaquine failure and subsequent relapse. The study sought to identify the association between CYP2D6 phenotype and recurrence of malaria in Korean patients.

**Methods:**

A total of 102 patients with vivax malaria were prospectively enrolled from eight institutions in Korea. An additional 38 blood samples from patients with recurred vivax malaria were provided by the Korea Disease Control and Prevention Agency. Malaria recurrence was defined as more than one episode of vivax malaria in the same or consecutive years. CYP2D6 star alleles, phenotypes, and activity scores were examined.

**Results:**

Genotyping for CYP2D6 was successful in 101 of the prospectively enrolled patients and 38 samples from the Korea Disease Control and Prevention Agency, of which 91 were included in the no-recurrence group and 48 were included in the recurrence group. Reduced CYP2D6 activity (intermediate metabolizer) phenotype was more common in the recurrence group than in the no-recurrence group (OR, 2.33 (95% CI, 1.14–4.77); *p* = 0.02). Patients with lower CYP2D6 activity had a higher probability of recurrence (*p* = 0.029).

**Conclusion:**

This study suggests that CYP2D6 polymorphism may affect primaquine efficacy and thus *Plasmodium vivax* recurrence in Korea.

**Supplementary Information:**

The online version contains supplementary material available at 10.1186/s12936-022-04311-6.

## Background

*Plasmodium vivax* is the only parasite that causes human malaria in South Korea. Since the re-emergence of *P. vivax* infection in 1993, the number of annual malaria cases peaked in the early 2000s and steadily decreased thereafter. However, malaria still occurs in around 400–500 patients annually in Korea and causes significant morbidities.

Unlike *Plasmodium falciparum*, *P. vivax* is known for its ability to cause recurrent clinical malaria from the activation of dormant liver stage parasites, known as hypnozoites. Currently, primaquine is the only approved hypnozoitocidal agent in Korea. Despite the use of primaquine, relapse of clinical malaria is reported in the range of 1.6–4.5% and is considered a major obstacle to the eradication of malaria in Korea [1–3]. The reason for the sub-optimal therapeutic efficacy of primaquine is complex. First, the drug is usually prescribed as a 14-day regimen in outpatient clinic, which hinders effective monitoring of drug compliance. Second, for patients with recurred malaria living in endemic areas, there is no standardized method for distinguishing relapse from re-infection.

Recently, metabolism of primaquine has been extensively studied to determine whether reductions in active metabolites may affect the anti-malarial properties of primaquine. Primaquine is metabolized into the active form via Cytochrome P450 isoenzyme 2D6 (CYP2D6) in the liver. CYP2D6 is a polymorphic gene with more than 149 known alleles that determine the CYP2D6 phenotype (i.e., poor, intermediate, normal, ultrarapid metabolizer) that has different levels of activity for drug metabolization [4]. Patients who are deemed to have poor or intermediate metabolic activity harbour impaired functions in the CYP2D6 enzyme; in such patients, even if an appropriate dose of primaquine is administered, a sufficient amount of active metabolites is not produced and may lead to therapeutic failure of primaquine in preventing recurrence [5, 6].

In this study, the association between the CYP2D6 phenotype and the risk of vivax malaria relapse in Korean patients was investigated.

## Methods

### Study sites and population

This study was performed at eight hospitals (National Health Insurance Service Ilsan Hospital, Inje University Ilsan Paik Hospital, Dongguk University Ilsan Hospital, Severance Hospital, Catholic Kwandong University International St. Mary’s Hospital, Asan Medical Center, Ajou University Hospital, and Armed Forces Capital Hospital) located in Gyeonggi Province and Seoul in South Korea, where malaria was endemic from 2018 to 2020.

Patients > 16 years of age diagnosed with vivax malaria during the study period were invited to participate in the study. Vivax malaria was diagnosed by peripheral blood smears (thick and thin smears stained with Giemsa stain) and/or polymerase chain reaction (PCR) analysis. Parasite density was determined by Giemsa-stained blood slides at a magnification of 1000× using WHO-recommended methods. Demographic information including age, gender and body weight were collected. Previous work has shown the prevalence of glucose-6-phosphate dehydrogenase (G6PD) deficiency in Koreans to be extremely low (near 0%) [7, 8], thus G6PD deficiency test was not performed prior to primaquine administration.

### Identification of recurred patients

Recurrence of malaria was identified by reviewing the medical records at each hospital and the National Infectious Disease Notification System. Malaria, indigenous or imported, is a mandatory notifiable disease in Korea and all individuals with malaria are reported to the Korea Diseases Control and Prevention Agency (KDCA) using the infectious disease reporting systems.

Malaria in Korea is seasonal, with 94% of cases occurring between 1993 and 2017 reported between May and October when malaria vectors are active. This seasonal distribution pattern of malaria cases has not changed over time. Thus, indigenous vivax malaria cases that occurred in the first four months of the year, winter and spring, might comprise *P. vivax* infections contracted in the previous malaria season that had a subsequent prolonged incubation, rather than new infection [9]. This characteristic epidemiology of Korean vivax malaria and prospective enrolled patients who had a notification record of another previous *P. vivax* malaria episode within the same year or the previous year were considered as recurred patients.

### Analysis of stored samples

Considering the low recurrence rate of vivax malaria in Korea (< 5%), there was an attempt to collect more blood samples of patients who were suspected of having recurred vivax malaria. Along with the disease notification to the KDCA, blood samples of most malaria patients are sent to KDCA for confirmation of malaria and further analysis. Accordingly, the blood samples of 38 patients who were suspected of having recurred vivax malaria with a record of a previous vivax malaria episode within the same year or the previous year between 2015 and 2018, as with the prospective enrolled patients, were provided by the KDCA and included in the analysis.

## CYP2D6 genotyping

CYP2D6 genotyping analysis was commissioned to SPMED Co. Ltd (Busan, South Korea). Patient DNA was isolated and purified from blood samples using SPMED™ Genotyping Kit: CYP2D6 (KFDA -IVD 20–297 and CE-IVD, SPMED Co. Ltd) according to the manufacturer’s instructions and CYP2D6 *2, *3, *4, *5 (deletion), *6, *9, *10, *14, *17, *18, *21, *29, *41, *49, *52, *60, *XN (duplicate) were analysed. These 17-star alleles cover most (99.3%) of the major mutation in Asian races based on previous results [10, 11].

Previously, full sequencing data from Korea have shown the *10 promoter and intron SNPs to be linked, such that the *10 alleles detected above is the CYP2D6*10b allele [12] and remains a reduced function allele.

Briefly, genomic DNA was extracted according to the manufacturer’s instructions of QIAgen Mini Kit (QIAamp Company’s DNA Mini Kit, Germany). The extracted DNA was stored at − 20 °C. PCR reactions were performed with 0.5 µl genomic DNA, 10 µl of PCR amplification primer mix, 4 µl of each CYP2D6 amplification primer mix. The total volume was adjusted to 20 µl with nuclease-free water. The PCR reaction protocol was controlled with an initial denaturation step at 94 °C for 5 min, followed by 35 cycles with first set at 98 °C for 20 s, 64 °C for 30 s, and 72 °C for 30 s, and a final extension reaction at 72 °C for 5 min. The amplified products were separated based on 1% agarose gel electrophoresis and visualized by ethidium bromide under a UV transilluminator. Only the sequences that showed identical size and 90% or more similar thickness with the wild type DNA were used for subsequent analysis.

Specific primers were used for single-base extension using SNaPshot kit (Applied Biosystems, Mannheim, Germany), according to manufacturer’s instructions. Reactions were carried out in a final volume of 13.5 µl, containing 1 µl of the SNaPshot Multiplex Reagent, 1 µl of the primer mix, 7.5 µl of purified product and 4 µl of 1/2 term buffer. Purified products underwent capillary electrophoresis on 3500 DX Series Genetic Analyzer (Applied Biosystems, Foster City, CA, USA) using the standard fragment analysis protocol.

GeneMapper software (version 4.0 Thermo Scientific, MA, USA) was used for allele-specific gene expression analysis and calculated the peak color of CYP2D6 single-nucleotide variant (SNV) to the peak density of the wild type DNA. The CYP2D6*1 allele was set when no nucleotide change was observed in all genotyped SNPs.

CYP2D6 genotype identified levels of enzymatic activity qualitatively by the activity score model, where a score of 0.0, 0.25–1.0, 1.25–2.25, and > 2.25 each indicate a poor metabolizer (PM), intermediate metabolizer (IM), normal metabolizer (NM), and ultrarapid metabolizer (UM), respectively [13].

### Statistical analysis

Descriptive statistics are expressed as means, medians or proportions. The associations between categorical data were tested using the χ-squared test or Mann-Whitney *U* test. For analysing the trends between categorical variables, the Cochran-Armitage test was used. All tests of significance were two-tailed, and differences were considered statistically significant at *p* < 0.05. All statistical analyses were performed using SPSS Statistics version 23 (IBM Corp., Armonk, NY, USA; https://www.ibm.com).

### Ethics statement

This study was approved by the ethical committees of each hospital (Asan Medical Centre: 2018-0650, Armed Forces Capital Hospital: AFCH 19-IRB-018, Dongguk University Ilsan Hospital: 2019-08-004-002, Inje University Ilsan Paik Hospital: 2019-07-020-001, National Health Insurance Service Ilsan Hospital: 2019-07-022-001, Severance Hospital: 4-2019-1240, Catholic Kwandong University International St. Mary’s Hospital: 19-IRB-059-1, Ajoo University Hospital: BMR-SMP-19-302). Signed written informed consents were obtained from prospectively registered patients or their guardians before the study. A waiver of patient consent was granted for data that were retrospectively collected.

## Results

A total of 102 patients with vivax malaria (not recurred, n = 92 (90.2%); recurred, n = 10 (9.8%)) were prospectively enrolled in the study, including one patient who had four episodes of malaria attack. Table 1 shows the demographic and clinical characteristics of the patients according to the presence of recurrence. The mean age of the total patients was 43.4 years and 75.8% were male. There were no statistically significant differences in age, gender distribution, or body weight between the two groups. Additional file 1: Table S1 shows the diagnostic date for each episode for 10 prospectively enrolled relapsed patients.

**Table 1:** Demographic and clinical characteristics of the prospectively enrolled patients according to recurrence

Variable	No recurrence (n = 92)	Recurrence (n = 10)	*P* value
Age, years (mean ± SD)	43.2 ± 17.5	46.0 ± 12.1	0.30
Male sex (%)	78.0	50.0	0.09
Body weight, kg (mean ± SD)	73.3 ± 12.3	73.8 ± 16.0	0.97
Initial parasitaemia, /µl (mean ± SD)	5,460.0 ± 7,403.1	6,568.0 ± 7,637.0	0.40
< 2000/µl (%)	41.0	100	
2000–20,000/µl (%)	56.4	0	
> 20,000/µl (%)	2.6	0	

CYP2D6 analysis was not available in one patient in the recurred group, and thus 101 patients underwent CYP2D6 analysis, whose CYP2D6 genetic profiles, activity score, phenotypes, and recurrence status are summarized in Table 2. A total of 38 samples of recurred patients from the KDCA were also retrospectively analysed, and Table 3 summarizes their CYP2D6 genetic profiles, activity score and phenotypes. Accordingly, a total of 139 samples were included in the analysis (no recurrence, n = 91 (65.5%); recurrence, n = 48 (34.5%)). The detailed information of SNPs for each CYP2D6 allele are shown in Additional file 2: Table S2. In the samples as a whole, a total of 16 CYP2D6 alleles were observed and the most common genotype was *10B/*10B, which was identified in 36 samples, including one patient who had four episodes of malaria attack (Table 4). The NM phenotype was the most common (n = 73 (52.5%)), followed by IM (n = 65 (46.8%)); UM phenotype was found in one patient and none had the PM phenotype (Table 5).

**Table 2:** Cytochrome P450 2D6 genetic profiles, activity score, phenotype, and recurrence status in the prospectively enrolled patients

Year	Genotype	Activity score	Phenotype	Recur	Year	Genotype	Activity score	Phenotype	Recur
2018	*5/*10B	0.25	IM	No	2019	*1/*1	2	NM	No
2018	*10B/*10B	0.5	IM	No	2019	*1/*2	2	NM	No
2018	*1/*10B	1.25	NM	No	2019	*1/*1	2	NM	No
2018	*10B/*10B	0.5	IM	No	2019	*10B/*10B	0.5	IM	No
2018	*1/*10B	1.25	NM	No	2019	*1/*10B	1.25	NM	Yes
2018	*10B/*49	0.75	IM	No	2019	*1XN/*5	2	NM	No
2018	*10B/*10B	0.5	IM	No	2019	*10B/*10B	0.5	IM	Yes
2018	*10B/*10B	0.5	IM	No	2019	*2/*41	1.5	NM	No
2018	*5/*10B	0.25	IM	No	2019	*1/*10B	1.25	NM	No
2018	*1/*1	2	NM	No	2019	*1/*2	2	NM	No
2018	*2/*10B	1.25	NM	No	2019	*1/*10B	1.25	NM	No
2018	*10B/*10B	0.5	IM	No	2019	*1/*10B	1.25	NM	No
2018	*5/*10B	0.25	IM	Yes	2019	*1/*10B	1.25	NM	No
2018	*1/*10B	1.25	NM	No	2019	*1/*21B	1	IM	No
2018	*1/*10B	1.25	NM	No	2019	*1/*10B	1.25	NM	No
2018	*5/*10B	0.25	IM	No	2019	*10B/*10B	0.5	IM	No
2018	*1/*10B	1.25	NM	No	2019	*1/*10B	1.25	NM	No
2018	*1/*41	1.5	NM	No	2019	*1/*5	1	IM	No
2018	*1/*2	2	NM	Yes	2019	*1/*10B	1.25	NM	No
2018	*10B/*10B	0.5	IM	No	2019	*2/*10B	1.25	NM	No
2018	*2/*41	1.5	NM	No	2019	*10B/*10B	0.5	IM	No
2018	*10B/*10B	0.5	IM	No	2019	*2/*5	1	IM	No
2018	*5/*14B	0.5	IM	No	2019	*1/*10B	1.25	NM	No
2018	*1/*10B	1.25	NM	No	2019	*10B/*10B	0.5	IM	Yes
2018	*2/*14B	1.5	NM	Yes	2019	*1/*2	2	NM	No
2018	*1/*2	2	NM	No	2019	*1/*1	2	NM	No
2018	*10B/*10B	0.5	IM	No	2019	*1/*1	2	NM	No
2018	*1/*10B	1.25	NM	No	2019	*1/*10B	1.25	NM	No
2018	*4/*10B	0.25	IM	Yes	2019	*5/*10B	0.25	IM	No
2018	*10B/*10B	0.5	IM	No	2019	*1/*10B	1.25	NM	No
2018	*10B/*10B	0.5	IM	No	2019	*10B/*10B	0.5	IM	No
2018	*10B/*10B	0.5	IM	No	2019	*2/*10B	1.25	NM	No
2018	*1/*10B	1.25	NM	No	2019	*1/*1	2	NM	No
2018	*10B/*10B	0.5	IM	No	2019	*10B/*10B	0.5	IM	No
2018	*1/*2	2	NM	No	2020	*10B/*10B	0.5	IM	No
2018	*1/*10B	1.25	NM	No	2020	*10B/*10B	0.5	IM	No
2018	*1/*10B	1.25	NM	No	2020	*1/*1	2	NM	No
2018	*2/*5	1	IM	No	2020	*1/*10B	1.25	NM	No
2018	*2/*10B	1.25	NM	No	2020	*10B/*41	0.75	IM	No
2018	*10B/*52	0.5	IM	Yes	2020	*10B/*10B	0.5	IM	No
2018	*1/*1	2	NM	No	2020	*5/*10B	0.25	IM	No
2018	*1/*10B	1.25	NM	No	2020	*2/*10B	1.25	NM	No
2018	*1/*14B	1.5	NM	No	2020	*2/*2	2	NM	No
2018	*1/*41	1.5	NM	No	2020	*1/*1	2	NM	No
2018	*10B/*10B	0.5	IM	No	2020	*10B/*10B	0.5	IM	Yes
2018	*10B/*10B	0.5	IM	No	2020	*1/*10B	1.25	NM	No
2018	*1/*2	2	NM	No	2020	*1/*2XN	3	UM	No
2018	*5/*10B	0.25	IM	No	2020	*1/*2	2	NM	No
2018	*1/*10B	1.25	NM	No	2020	*1/*21B	1	IM	No
2019	*10B/*10B	0.5	IM	Yes	2020	*1/*41	1.5	NM	No
2019	*1/*10B	1.25	NM	No					

*IM* intermediate metabolizer, *NM* normal metabolizer, *PM* poor metabolizer, *UM* ultrarapid metabolizer

**Table 3:** Cytochrome P450 2D6 genetic profiles, activity score and phenotypes in the retrospectively collected 38 samples from recurred patients

Year	Genotype	Activity score	Phenotype	Year	Genotype	Activity score	Phenotype
2015	*2/*41	1.5	NM	2017	*2/*10B	1.25	NM
2015	*10B /*21B	0.25	IM	2017	*10B/*10B	0.5	IM
2015	*1/*10B	1.25	NM	2017	*2/*10B	1.25	NM
2015	*10B/*10B	0.5	IM	2017	*10B/*10B	0.5	IM
2015	*1/*1	2	NM	2017	*1/*10B	1.25	NM
2015	*2/*10B	1.25	NM	2017	*10B/*10B	0.5	IM
2015	*1/*10B	1.25	NM	2017	*10B/*10B	0.5	IM
2016	*1/*5	1	IM	2017	*10B/*41	0.75	IM
2016	*1/*5	1	IM	2017	*2/*10B	1.25	NM
2016	*1/*1	2	NM	2018	*10B/*52	0.5	IM
2016	*5/*41	0.5	IM	2018	*1/*10B	1.25	NM
2016	*5/*10B	0.25	IM	2018	*10B/*10B	0.5	IM
2016	*5/*10B	0.25	IM	2018	*2/*10B	1.25	NM
2016	*1/*10B	1.25	NM	2018	*10B/*10B	0.5	IM
2016	*1/*5	1	IM	2018	*1/*2	2	NM
2016	*10B/*10B	0.5	IM	2018	*1/*10B	1.25	NM
2016	*1/*5	1	IM	2018	*4/*10B	0.25	IM
2016	*10B/*10B	0.5	IM	2018	*1/*5	1	IM
2016	*1/*10B	1.25	NM	2018	*10B/*10B	0.5	IM

**Table 4:** The summarization of number of each genotypes for all patients

Genotype	Frequency (%)	Genotype	Frequency (%)
*1/*1	11 (7.9)	*4/*10B	2 (1.4)
*1/*2	9 (6.5)	*5/*10B	9 (6.5)
*1/*2xN^†^	1 (0.7)	*5/*41	1 (0.7)
*1/*5	6 (4.3)	*5/*14B	1 (0.7)
*1/*10B	33 (23.7)	*10B/*10B	36 (25.9)
*1/*14B	1 (0.7)	*10B/*21B	1 (0.7)
*1/*21B	2 (1.4)	*10B/*41	2 (1.4)
*1/*41	3 (2.2)	*10B/*49	1 (0.7)
*1xN^††^/*5	1 (0.7)	*10B/*52	2 (1.4)
*2/*2	1 (0.7)		
*2/*5	2 (1.4)		
*2/*10B	10 (7.2)		
*2/*14B	1 (0.7)		
*2/*41	3 (2.2)		

^†^The phenotype of CYP2D6*2XN maintain UM even if the CYP gene duplicated or more

^††^The phenotype will be IM if CYP2D6*1XN gene is duplicated, and the phenotype changes to UM if multiplicated. According to the CYP2D6 frequency and CYP2D6 Allele Functionality Table (https://www.pharmgkb.org/page/cyp2d6RefMaterials), however, in East Asians, including Koreans, triplication or more copies are rarely reported, so it is generally assumed as duplication

**Table 5:** The summarization of number of each phenotype for all patients

Phenotype	Frequency (%)
PM	0 (0)
IM	65 (46.8)
NM	73 (52.5)
UM	1 (0.7)

In line with previous studies, CYP2D6 phenotypes were classified and analysed into two categories: PM + IM vs. NM + UM. Recurrence was more common in the PM + IM group than the NM + UM group, with an OR of 2.33 (95% CI, 1.14–4.77, *p* = 0.02) (Table 6).

**Table 6:** Association of vivax malaria recurrence with the cytochrome P450 2D6 phenotype

Genotype	No recurrence (n = 91)	Recurrence (n = 48)	*P* value
Poor or intermediate metabolizer	36 (39.6%)	29 (60.4%)	0.02
Normal or ultrarapid metabolizer	55 (60.4%)	19 (39.6%)	

Data are n (%)

The activity scores of the total patients ranged from 0.25 to 3, with 108 (77.7%) patients showing a score lower than 1.5 and 31 (22.3%) patients showing a score of 1.5 or higher. When the association between recurrence and the activity scores was analysed, patients with higher activity scores were significantly less likely to have a recurrence (*p* = 0.028) (Table 7).

**Table 7:** Association of vivax malaria recurrence with the cytochrome P450 2D6 activity score

Activity score	0.25	0.5	0.75	1.0	1.25	1.5	2	3	Total
Number of patients	12	40	3	10	43	8	22	1	139
Recurred patients	6	17	1	5	13	2	4	0	48
Proportion of recurrence (%)	50.0	42.5	33.3	50.0	30.2	25.0	18.2	0.2	

*The Cochran-Armitage test for trends were statistically significant (*p* = 0.029)

## Discussion

In this study, the association of the recurrence of *P. vivax* infection with CYP2D6 phenotypes, which affects the metabolism of primaquine, was investigated. Patients who were deemed as PM or IM, as determined by the CYP2D6 phenotype, had a significantly higher risk of recurrence than those deemed as NM or UM. Those who had lower genotype-determined activity scores had a higher recurrence risk than those with higher activity scores. These findings suggest that malaria may recur in patients with low CYP2D6 enzyme activity that hampers primaquine treatment due to decreased production of its active metabolite.

The findings support previous studies that suggested the association of CYP2D6 phenotype and the risk of relapse in patients with vivax malaria [5, 6, 14–16]. These studies were performed in various geographical areas and included patients from Indonesia, Brazil, Malaysia, and Spain. This current study on Korean patients also reached a similar conclusion. Although some studies did not find a significant association between CYP2D6 and recurrence of *P. vivax* [17], this study adds valuable evidence for the role of CYP2D6 in the efficacy of primaquine.

The CYP2D6 allele frequencies observed in the current study were similar to those reported in previous studies performed in Korea [18, 19]. The *1, *2, *10B alleles were the most common alleles in this study, comprising 29.1, 11.2 and 47.5% of the allele frequency, respectively. Specifically, one study on CYP2D6 found that approximately 40% of Southeast Asians possess the CYP2D6 *10 allele [20].

In terms of the genotype, the most common genotype was also *10B/*10B (36/139 (25.9%)) in this study. Genotypes *1/*10B, *1/*1, and *2/*10B were found in 23.7, 7.9 and 7.2%, respectively. These genotypes were also common in other Asian countries, such as China, Japan and Thailand [21]. The genotype-to-phenotype translation of CYP2D6 was recently reclassified and standardized [13], and this new classification system was adapted in the current study. Accordingly, it was found that 46.7% (65/139) had the IM phenotype and none had the PM phenotype.

In addition to the CYP2D6 phenotype, many factors could affect the efficacy of primaquine. The total dose of primaquine must be considered by priority. In Korea, primaquine 15 mg for 14 days had been traditionally recommended [22]. However, since the average body weight of Koreans has continuously increased in recent years, it is often pointed out that the recommended dose of primaquine could be insufficient in contemporary patients. The dosages of primaquine administered to each patient of the samples provided by KCDA could not be confirmed. For 102 prospectively registered patients, however, primaquine was administered from the next day after three days of chloroquine treatment and usually for 14 days. Fifteen mg/day of primaquine was administered for patients weighing 70 kg or less, and 30 mg/kg of primaquine was administered for patients weighing more than 70 kg. The primaquine dosage at the second attack was confirmed in all but two prospectively registered patients and the average daily dose of was 0.31 ± 0.09 mg/kg per day, suggesting the proportion of underdosing was not high. Whether this increased dosage of primaquine could overcome the effect of decreased CYP2D6 enzyme activity is not certain. Further studies on the optimal dosage and duration of primaquine are needed.

One limitation of this study is that detailed information could not be obtained on other drugs that the patients may have used. Drug-drug interaction could significantly affect the metabolism of primaquine, and the CYP2D6 enzyme is known to be inhibited by commonly used drugs such as fluoxetine, duloxetine and terbinafine [23]. Thus, it should be noted that patients who take these medications could have relatively decreased CYP2D6 activity, which could lower the effect of primaquine. Another limitation is that patients with recurrent *P. vivax* infection, which includes relapse, reactivation of the hypnozoites, and re-infection were included. Several studies identified that the molecular markers of *P. vivax* could be either homogenous or heterogeneous between the primary episode and relapsed episodes [24, 25]. Currently, there is no reliable method to differentiate between relapse and re-infection. One patient with an IM phenotype in the study reported four episodes of malaria attack within one year, and the molecular analysis of the four samples showed identical genetic profiles, suggesting a relapse of the homologous clone rather than re-infection. Thirdly, no additional copy number analysis was performed for the samples in which CYP2D6*1XN and CYP2D6*2XN was found. However, the phenotype of CYP2D6*2XN is maintained UM even if the CYP gene duplicated or more. The phenotype will be IM if CYP2D6*1XN gene is duplicated, and the phenotype changes to UM if multiplicated. However, in East Asians including Koreans, triplication or more copies are rarely reported, so it is generally assumed as duplication [26].

## Conclusion

This study found that patients deemed as intermediate metabolizers as determined by CYP2D6 enzyme activity had a higher risk of vivax malaria recurrence than did those deemed as normal or ultrarapid metabolizers. CYP2D6-dependent metabolism of primaquine may be a key determinant of the anti-hypnozoite activity of primaquine that prevents vivax malaria relapse. Additional study for the optimization of primaquine efficacy is urgently needed.

## Supplementary information

**Additional file 1**: **Table S1. **Diagnostic date for each episode of vivax malaria for 10 prospectively enrolled relapsed patients.

**Additional file 2**: **Table S2. **The CYP2D6 alleles of the entire samples defined by tagging single nucleotide polymorphisms (tSNPs).

## Data Availability

The datasets used and/or analysed during the current study are available from the corresponding author on reasonable request.

## Abbreviations

CYP2D6: Cytochrome P450 isoenzyme 2D6
PCR: Polymerase chain reaction
KDCA: Korea Diseases Control and Prevention Agency
PM: Poor metabolizer
IM: Intermediate metabolizer
NM: Normal metabolizer
UM: Ultrarapid metabolizer
G6PD: Glocose-6-phosphate dehydrogenase
SNV: Single nucleotide variant
SNP: Single nucleotide polymorphism
