# Supplementary material for: Association between CYP2D6 phenotype and recurrence of Plasmodium vivax infection in south Korean patients
Source: Malar J. 2022 Oct 10;21:289. doi: 10.1186/s12936-022-04311-6 (PMC9552356; doi:10.1186/s12936-022-04311-6)
Supplement: Supplementary file 1 — Additional file 1: Table S1. Diagnostic date for each episode of vivax malaria for 10 prospectively enrolled relapsed patients. [file 12936_2022_4311_MOESM1_ESM.docx]

**Supplementary Table 1**  **Diagnostic date for each episode of vivax malaria for 10 prospectively enrolled relapsed patients**

| **Patients No.** | **Diagnostic date (*P. vivax* PCR positive)** | | | |
| --- | --- | --- | --- | --- |
|  | 1^st^ attack | 2^nd^ attack | 3^rd^ attack | 4^th^ attack |
| Patient 1 | September, 2017 | July, 2018 |  |  |
| Patient 2 | August, 2017 | July, 2018 |  |  |
| Patient 3 | September, 2017 | July, 2018 | August, 2018 |  |
| Patient 4 | September, 2017 | July, 2018 |  |  |
| Patient 5 | February, 2018 | August, 2018 |  |  |
| Patient 6 | March, 2019 | April, 2019 | May, 2019 | September, 2019 |
| Patient 7 | July, 2019 | August, 2019 |  |  |
| Patient 8 | Summer, 2018^*^ | April, 2019 |  |  |
| Patient 9 | June, 2018 | April, 2019 | September, 2019 |  |
| Patient 10 | April, 2020 | Jun, 2020 |  |  |

^*^ This case was not possible to ascertain the exact month.

**Supplementary Table 2 The CYP2D6 alleles of the entire samples defined by tagging single nucleotide polymorphisms (tSNPs)**
